# Visualization of exosome-mediated miR-210 transfer from hypoxic tumor cells

**DOI:** 10.18632/oncotarget.14247

**Published:** 2016-12-27

**Authors:** Kyung Oh Jung, Hyewon Youn, Chul-Hee Lee, Keon Wook Kang, June-Key Chung

**Affiliations:** ^1^ Department of Nuclear Medicine, Seoul National University College of Medicine, Seoul, Korea, 110-799; ^2^ Department of Biomedical Sciences, Seoul National University College of Medicine, Seoul, Korea, 110-799; ^3^ Cancer Research Institute, Seoul National University College of Medicine, Seoul, Korea, 110-799; ^4^ Cancer Imaging Center, Seoul National University Hospital, Seoul, Korea, 110-799; ^5^ Department of Radiation Oncology & Medical Physics, Stanford University, CA, USA, 94305

**Keywords:** exosome, breast cancer, miR-210, tumor microenvironment, hypoxia

## Abstract

Cancer cells actively release exosomes carrying specific cellular components, such as proteins, mRNA, and miRNA, to communicate with various cells in the tumor microenvironment. We visualized exosome-mediated transfer of miR-210 from hypoxic breast cancer cells to neighboring cells using a miR-210 specific reporter system. By *in vitro* and *in vivo* visualization, we found that exosomes with miR-210 were transferred to cells in the tumor microenvironment and that miR-210 was involved in expression of vascular remodeling related genes, such as Ephrin A3 and PTP1B, to promote angiogenesis. These results indicate that cellular components, such as miRNAs from hypoxic cancer cells, spread to adjacent cancer cells in the tumor microenvironment via exosomes and influence tumor progression.

## INTRODUCTION

Hypoxia is one of the hallmarks of cancer [1, 2]. Cells within the tumor become hypoxic as the tumor mass increases, resulting in activation of HIF-1α to induce various malignant phenotypes. Hypoxic tumors become resistant to chemotherapy and radiotherapy; therefore, imaging hypoxia is important in cancer diagnostics and therapeutic planning [3, 4].

Recently, some microRNAs have been considered cancer biomarkers because of their important roles in regulating gene expression [5]. MicroRNAs are single-stranded, non-coding small RNAs that regulate degradation or post-translational inhibition of target mRNA by partially or completely binding to the 3′ untranslated region (UTR) of the mRNA [6]. Among the possible cancer biomarker candidates, miR-210 levels have been reported to be highly increased in hypoxic cells and may be involved in tumor growth and angiogenesis [7, 8].

As microvesicles (30–100 nm in diameter) secreted by cells, exosomes contain many functional proteins, mRNAs, and miRNAs, and play a role in intercellular communication [9, 10]. Recently, the roles of exosomes in the tumor microenvironment have been emphasized for their involvement in tumor progression, angiogenesis, and metastasis [11–13]. Several studies have shown that miR-210 levels in hypoxic cells and the relative amount of secreted exosomes from hypoxic cancer cells are highly increased [14, 15]. However, exosome-mediated transfer of miR-210 to neighboring cells and the effects of hypoxic exosomes on the tumor microenvironment have not been clearly elucidated.

In this study, we constructed a reporter gene vector with triple seed sequences to visualize the presence of miR-210 and investigated the effect of exosome-mediated transfer of miR-210 in the tumor microenvironment using reporter expressing cells.

## RESULTS

### Characterization of exosomes and *in vivo* biodistribution

**Figure 1:**
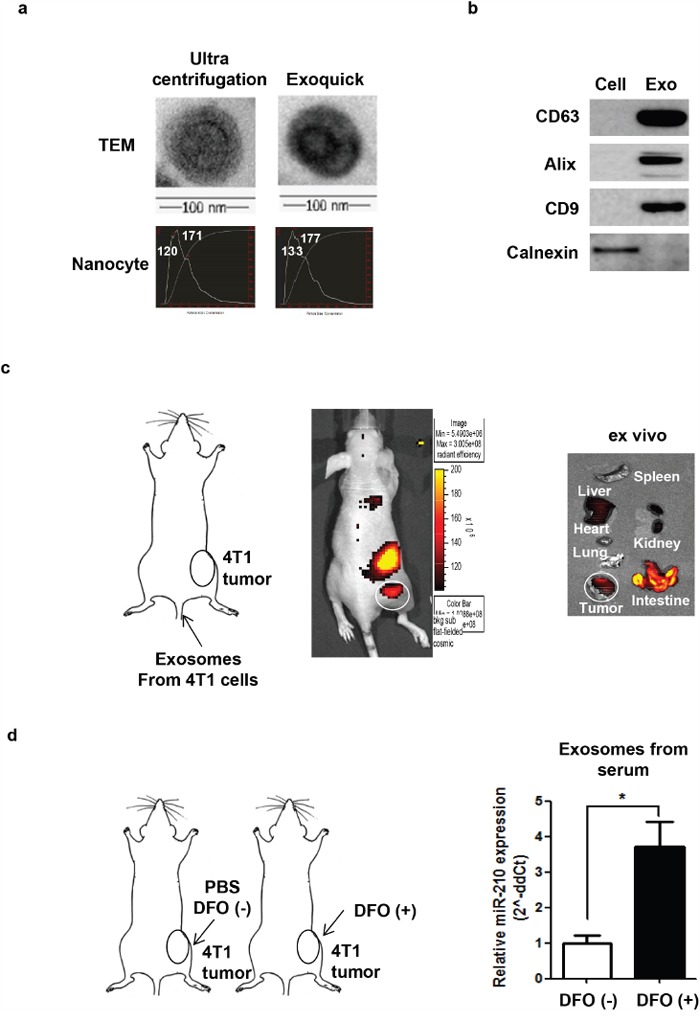
Biodistribution of exosomes and DFO-induced exosomal miR-210 expression in 4T1 tumor-bearing mice **a**. Characterization of exosomes with TEM imaging and Nanocyte. Exosomes were purified from the culture medium using ultracentrifugation and ExoQuick^™^. **b**. Characterization of exosomes with western blot analysis. Exosome marker proteins (CD9, CD63, and Alix); cell marker protein (calnexin). **c**. Localization of Cy7-labeled exosomes in 4T1 tumor bearing mice. **d**. Induction of exosomal miR-210 in serum from DFO (+) mice. *P <0.05.

To characterize exosomes, ultra-centrifugation and the ExoQuick kit were used for purification. Transmission Electron Microscopy and NanoSight revealed the presence of microvesicles from both isolation methods, and their morphology and size were within the expected range of an exosome (∼100 nm) (Figure 1a). Purified exosomes had exosome markers, including CD9, CD63, and Alix. The cellular marker calnexin was not observed (Figure 1b).

To visualize the biodistribution of exosomes in mice *in vivo*, we isolated exosomes from 4T1 cells, labeled them with Cy7 fluorescent dye, and collected images after intravenous injection into 4T1 tumor models. Cy7-exosomes (Supplementary Figure 1) accumulated in 4T1 tumors *in vivo* and *ex vivo* (Figure 1c). To investigate systemic transfer of exosomal miR-210 in the blood circulation, we directly injected DFO (200 μM) into 4T1 tumor grafts in mice and isolated exosomes from the serum. We observed a significant increase in the amount of miR-210 from exosomes isolated from the serum of DFO-treated mice (3.71-fold; P = 0.0368), indicating systemic circulation of exosomes containing miR-210 (Figure 1d).

Because we used DFO to induce hypoxia, we evaluated the cytotoxicity of DFO in 4T1 cells (Supplementary Figure 2a). Less than 400 μM of DFO was considered non-toxic to 4T1 cells.

### DFO-induced hypoxia and exosomes in a hypoxic environment

**Figure 2:**
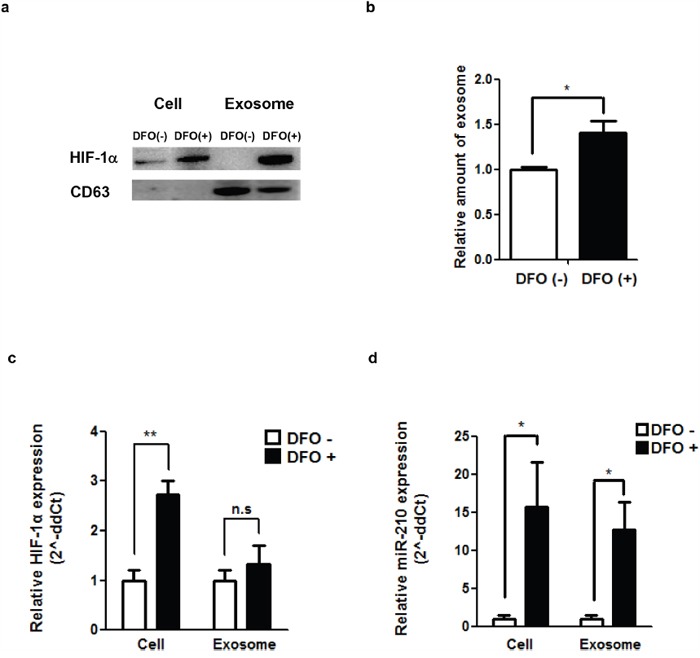
DFO-induced hypoxia and exosomes in a hypoxic environment **a**. Induction of a hypoxic environment with DFO (400 μM, 48 hr treatment) and the amount of Hif-1α as evaluated by western blot. **b**. Relative amount of exosomes produced by 4T1 cells after treatment with DFO. **c**. HIF-1α expression in cells and exosomes after treatment with DFO. **d**. miR-210 expression in cells and exosomes after DFO treatment. *P <0.05.

To compare DFO-mediated hypoxia and natural hypoxia, we measured HIF-1α levels with and without DFO treatment. HIF-1α protein levels were increased in DFO treated (+) cells and exosomes compared to the control (Figure 2a). The amount of secreted exosomes in hypoxic cells was measured from the culture media of 4T1 cells with or without DFO treatment. Increased exosome secretion was observed in the medium of DFO (+) cells compared to DFO (-) cells (1.40-fold, P = 0.0047, Figure 2b).

Based on real-time quantitative PCR (Figure 2c), cellular HIF-1α expression levels in DFO (+) cells were increased after DFO treatment compared with DFO (-) cells (2.73-fold, P=0.00403). However, there was no significant difference in exosomal HIF-1α levels regardless of DFO treatment. Cellular and exosomal miR-210 levels in DFO (+) cells showed a significant increase (Figure 2d) compared with DFO (-) cells (15.70-fold, P = 0.0184 for cellular miR-210; 12.73-fold, P = 0.0023 for exosomal miR-210).

### Imaging miR-210 activation in hypoxic cancer cells

**Figure 3:**
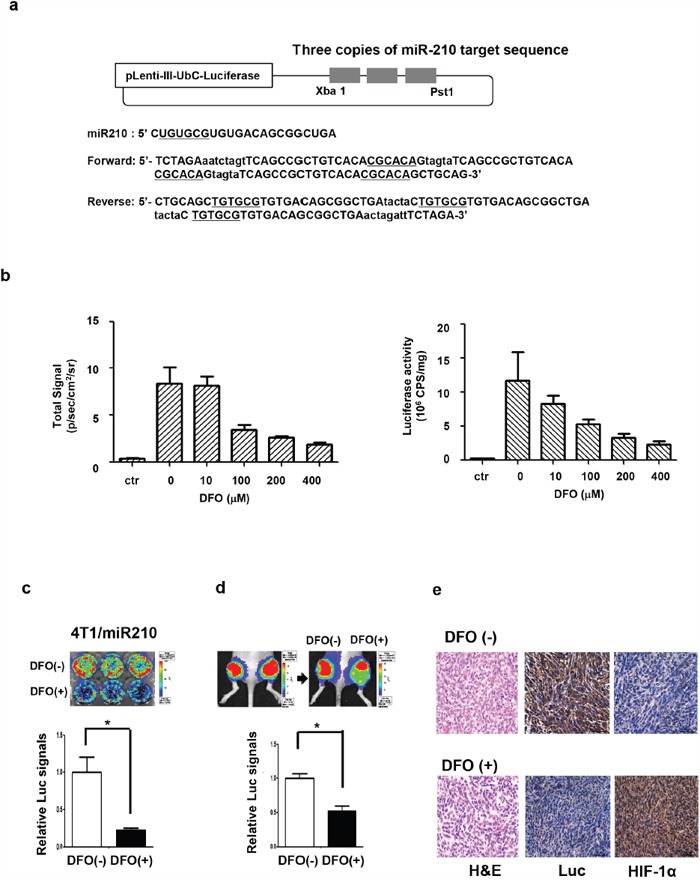
Imaging miR-210 expression by DFO-induced hypoxia in 4T1 cells **a**. Design of miR-210 reporter construct to image miR-210 expression. 4T1-luc2/miR-210 cells that express a miR-210 reporter vector were established, and luciferase signals from these cells could be turned off by the binding of miR-210. **b**. Reporter activity assay in 4T1 cells. IVIS images and luciferase assays showed that luciferase signals (left) and luciferase activity (right) were decreased by DFO treatment in a dose-dependent manner. **c**. Bioluminescence imaging showed that luciferase (Luc) signals from 400 μM of DFO-treated cells were significantly decreased. **d**. Bioluminescence imaging of 4T1/miR-210 tumor-bearing mice; luciferase signals from the DFO (+) tumor were significantly decreased (n=3). DFO (400 μM) was directly injected into the 4T1/miR-210 tumor. **e**. Immunohistochemistry of the DFO (+/-) 4T1/miR-210 tumor. Luciferase expression in DFO (+) tumors was decreased compared to DFO (-) tumors, whereas HIF-1α expression in DFO (+) tumors was increased compared to DFO (-) tumors. *P<0.05.

To visualize miR-210 in cells, we designed a luciferase-based miR-210 reporter vector that contains three repeated miR-210 target sequences (CGCACA) to amplify sensitivity to miR-210 binding (Figure 3a). In this reporter system, miR-210 binding can turn off the luciferase signal due to formation of double stranded RNA. To test the miR-210 reporter function of this vector, we established a 4T1 cell line expressing a miR-210 reporter (4T1/miR210). We induced miR-210 with DFO treatment in the reporter expressing cells and evaluated luciferase activity. Both signals from IVIS imaging and the luciferase activity determined from an enzymatic assay were decreased in a DFO dose-dependent manner (Figure 3b). In bioluminescent imaging of cells (Figure 3c), signals from DFO (+) cells were decreased compared to those from DFO (-) cells (0.22-fold, P = 0. 0057). For *in vivo* imaging, DFO was injected directly into the 4T1/miR210 tumor in mice. Luciferase signals from the tumor were decreased after DFO treatment, whereas signals from tumors treated with PBS as a control were similar before and after treatment. Luciferase signals from DFO (+) cells were decreased by 0.53-fold (P = 0.0271) compared to those from DFO (-) cells (Figure 3d). From IHC of tissues (Figure 3e), we observed that luciferase expression in DFO (+) tumors was decreased and HIF-1α expression in DFO (+) tumors was increased compared to DFO (-) tumors.

### Imaging uptake of exosomes by cells in the tumor microenvironment

To confirm uptake of exosomes in the tumor microenvironment, exosomes were labeled with DiI/or DiO and imaged with the Maestro™ fluorescence imaging system and confocal microscopy (Supplementary Figure 3a). Fluorescence-labeled exosomes were exposed to various cells in the tumor microenvironment, such as tumor cells (4T1), endothelial cells (SVEC), macrophages (Raw264.7), stem cells (mBs-MSC), fibroblasts (3T3), and dendritic cells (JAWS2). In confocal microscopy, uptake of fluorescently stained exosomes in various cells was also observed (Supplementary Figure 3b). To image exosomes using another method, we constructed a CMV-driven GFP/RFP-tagged CD9 vector using the well-known exosomal marker protein CD9. In both confocal microscopy and Maestro images, we were able to image the fluorescence expressing exosomes in the CD9-GFP/RFP vector-transfected 4T1 cells (Supplementary Figure 4a, 4b).

### Imaging of exosome-mediated transfer of miR-210 to recipient cancer cells *in vitro* and *in vivo*

**Figure 4:**
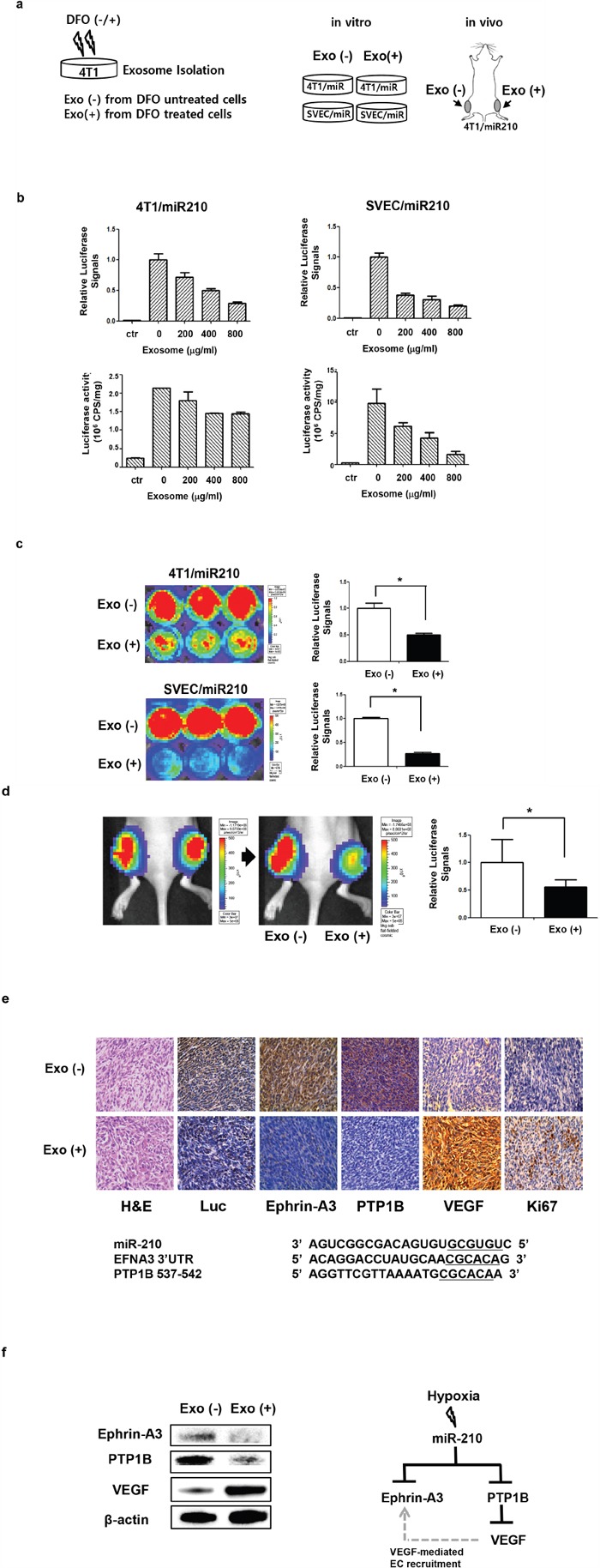
*In vitro* and *in vivo* miR-210 activation by hypoxic exosomes **a**. Experimental scheme for imaging miR-210 activation by DFO-induced hypoxic exosomes. Exosomes from DFO-treated 4T1 cells were designated as EXO (+), and exosomes from PBS-treated 4T1 cells were designated as EXO (-). **b**. Both bioluminescence images and the luciferase assay showed a dose-dependent decrease in luciferase activity in cells treated with hypoxic exosomes (Exo (+)). **c**. Bioluminescence imaging of 4T1/miR210 and SVEC/miR210 cells after treatment with exosomes from DFO-treated/or -untreated 4T1 cells. **d**. Bioluminescence imaging of the 4T1/miR210 tumor after intra-tumoral treatment with exosomes (400 μg) from DFO-treated or -untreated 4T1 cells (n=4). **e**. Immunohistochemistry of 4T1/miR210 tumor after treatment with exosomes. Expression of luciferase and miR-210 targets, such as Ephrin-A3 and PTP1B, was decreased in Exo (+) tumors, while expression of VEGF and Ki67 was increased in Exo (+) tumors. **f**. Western blotting of miR-210 target proteins and angiogenesis factor VEGF from 4T1/miR210 tumor tissues and their possible interactions. *P<0.05.

To assess miR-210 transfer through exosomes, we isolated exosomes from DFO-treated 4T1 cells (hypoxic exosomes) and added them to 4T1/miR-210 or SVEC/miR-210 cells for *in vitro* evaluation. We also injected exosomes from DFO-treated/or non-treated 4T1 cells (designated as EXO (+)/or EXO (-)) to grafted tumors with 4T1/miR-210 to visualize exosome-mediated transfer of miR-210 *in vivo* (Figure 4a). When we exposed hypoxic exosomes to 4T1/miR-210 and SVEC/miR-210 cells, IVIS images and luciferase activity assay showed that luciferase signals in both cells were significantly decreased in a dose-dependent manner (Figure 4b). Cytotoxicity of the exosome treatment was also evaluated in both 4T1 and SVEC cells (Supplementary Figure 2b, 2c). Even with an increased amount of exosomes, treatments were not cytotoxic to 4T1 and SVEC cells.

After 48 h treatment with exosomes, luciferase signals from Exo (+) 4T1 cells were decreased 0.67-fold (P = 0. 0262) compared to Exo (-) 4T1 cells (Figure 4c). Luciferase signals from Exo (+) SVEC cells were also significantly decreased 0.27-fold (P = 0. 0005) compared to Exo (-) SVEC cells. To measure the effect of hypoxic exosomes on SVEC cells, wound healing assays were performed. We observed that migration of Exo (+) SVEC cells was higher than in Exo (-) SVEC cells (Supplementary Figure 5a), and capillary-like structures were increased in Exo (+) SVEC cells (Supplementary Figure 5b). Furthermore, proliferation of Exo (+) SVEC cells was increased under a prolonged incubation time (Supplementary Figure 5c).

In bioluminescence imaging of grafted tumor models (Figure 4d), luciferase signals in the Exo (+) tumor were also decreased after exosome treatment, while signals in Exo (-) tumors were similar before and after treatment. From the ROIs, signals in Exo (+) tumors were significantly decreased (0.56-fold, P = 0. 0174), whereas signals in Exo (-) tumors did not decrease significantly. Tumor IHC showed that luciferase expression in Exo (+) tumors was decreased compared to Exo (-) tumors. Moreover, Ephrin-A3 and PTP1B expression, which are miR-210 target proteins, was also decreased in Exo (+) tumors compared to Exo (-) tumors (Figure 4e). However, VEGF and Ki67 levels were increased in Exo (+) tumors compared to Exo (-) tumors. Based on western blot analysis of tumor tissues (Figure 4f), we confirmed that results were similar to IHC findings and showed that Ephrin-A3 and PTP1B protein levels were reduced in Exo (+) tumors, while VEGF was increased in Exo (+) tumors.

## DISCUSSION

In this study, we developed a luciferase-based reporter vector to monitor miR-210. We demonstrated that exosomes from hypoxic tumor cells can transfer miRNA-210 to normoxic tumor/or endothelial cells and that exosomal miR-210 inhibited target genes and promoted angiogenesis in recipient cells.

Recently, exosomes have been actively investigated as novel messengers in cell-to-cell communication. The lipid bilayer of exosomes ensures the stability of their contents by protecting RNAs and proteins from degradation by circulating nucleases and proteases [16–19]. Because of their unique physiological characteristics, considerable attention has been given to the use of exosomes for diagnostic and therapeutic applications [20–22]. Fluorescence images of exosomes in a tumor-grafted mouse show localization of exosomes in tumors (Figure 1b), suggesting tumor tropism of exosomes. Because exosomes are bioavailable, well-tolerable, targetable, and membrane-permeable, they are ideal candidates for delivery of miRNA, proteins, drugs, and other molecules to tumors.

Hypoxia is an important feature in tumors with malignant phenotypes and poor prognoses [23, 24]. Hypoxic tumors may communicate with surrounding tumor and non-tumor cells through exosomes to induce phenotypes that are more malignant. In particular, miR-210, which is induced by hypoxia, is associated with tumor progression, angiogenesis, and metastasis [25]. Our data show that exosomes from the serum of hypoxic tumor-bearing mice had high miR-210 levels compared to normal mouse serum (Figure 1d). These results indicate that miR-210 from circulating exosomes in the serum can be used as a potential biomarker for hypoxic tumors. In particular, our results suggest that exosome-mediated systemic transfer of miR-210 could influence nearby cells to produce a more favorable environment for tumor survival.

These results also show that exosomal miR-210 from hypoxic cancer cells can be transferred to various types of recipient cells, such as epithelial cells, immune cells, and mesenchymal stem cells. It is possible that miRNA of exosomes from cancer cells can be transferred not only to surrounding cancer cells but also to neighboring stromal cells [29]. Functional efficiency of exosome-mediated miR-210 seems to be different in cell types. When we treated 4T1 and SVEC cells with the same amount of exosomes (Figure 4c), changes in luciferase signals between 4T1 and SVEC cells were quite different, suggesting that efficiency of exosome-mediated miR-210 functionality is dependent on cell type. The mode, effect, and clinical significance of exosome transfer to these microenvironment cells should be clarified.

Various target genes of miR-210, such as Ephrin-A3, PTP1B, HOXA1, and FGFRL1 [7], were reported, but we selected Ephrin-A3 and PTP1B because of their association with VEGF signaling and angiogenesis [26, 27]. Results show that Ephrin-A3 and PTP1B levels in tumors were decreased by treatment with exosomes containing miR-210. In contrast, VEGF levels in tumors were increased by treatment of exosomes with miR-210 (Figure 4e). Since Ephrin-A3 and PTP1B play important roles in the development of vascular remodeling [28], we speculated that downregulation of Ephrin-A3 and PTP1B through exosomal miR-210 could be correlated with angiogenic responses, resulting in formation of new capillaries and tubular structures.

**Figure 5:**
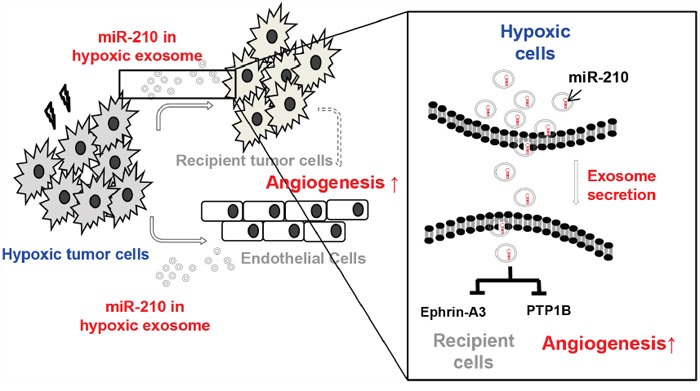
Summary. Hypoxic tumor cells release exosomes containing miR-210 and transfer them to neighboring cells in the tumor microenvironment Recipient cells showed inhibition of miR-210 target genes, such as Ephrin-A3 and PTP1B, which influence changes in vascular structure to promote angiogenesis.

Figure 5 shows that hypoxic tumor cells release miR-210 containing exosomes and that these exosomes are transferred to neighboring recipient cells. We found that transfer of exosomal miR-210 from hypoxic cells results in inhibition of miR-210 target genes, such as Ephrin-A3 and PTP1B, in neighboring cells in the tumor microenvironment. Vascular changes by down-regulation of Ephrin-A3 and PTP1B could increase VEGF and promote VEGF-mediated endothelial cell recruitment.

Although we only showed one example of exosome-mediated transfer of hypoxia-induced genetic material (miR-210), it is important to visualize exosome-mediated transfer of other hypoxia-induced miRs, such as miR-26, miR-31, miR107, and miR-424 [30–33] and their effects on the tumor microenvironment to investigate exosome-mediated malignant phenotype transfer of hypoxic cancer cells. Modifying imaging systems with other miRNA binding sites and monitoring cell-to-cell communication through exosomes will provide better knowledge to understand the role of exosomes in the tumor microenvironment.

## MATERIALS AND METHODS

### Establishment of stable cell lines and hypoxic exposure

A mouse breast cancer cell line (4T1) and endothelial cells (SVEC) were grown as monolayer cultures in RPMI-1640 medium supplemented with 1% antibiotic–antimycotic mix and 10% fetal bovine serum (FBS). Cells were infected by retroviruses with reporter gene vectors pCMV-luc2/miR-210 in which luciferase signals could be turned off by the binding of miR-210 to triplicates of the miR-210 binding site at the 3′ end of luc2 mRNA. Stable cell lines were selected by treatment with puromycin (2 g/mL) for 2 weeks. 4T1 cell lines with pCMV-luc2/miR-210 were labeled 4T1/miR210. Hypoxic conditions were induced with deferoxamine mesylate (DFO) (Sigma-Aldrich, St Louis, MO, USA) at 37°C in a 5% CO_2_ humidified environment.

### Exosome purification

4T1 cells (1 × 10^6^ cells) were cultured in conditioned medium. After 48 h, exosomes were isolated from the culture medium using ultracentrifugation (10,000 × *g*) or an exosome purification kit (ExoQuick^™^, System Bioscience, Mountain View, CA, USA). Exosome pellets were washed and resuspended with PBS. Proteins from exosome pellets and lysed cells were obtained by 1X RIPA buffer (25 mM Tris-HCl pH 7.6, 150 mM NaCl, 1% NP-40, 1% sodium deoxycholate, 0.1% SDS) with a cocktail of protease inhibitors (Roche, Nutley, NJ, USA). Protein concentrations were measured using a BCA protein assay kit (Pierce, Rockford, IL, USA).

### Transmission electron microscopy and NanoSight

Exosomes were fixed with 2% glutaraldehyde (GAA) overnight at 4°C and deposited by a copper grid (300 mesh covered with carbon). Size of exosomes was analyzed using transmission electron microscopy (TEM) images obtained using a JEOL (JEM 1400) transmission electron microscope at 80 keV and measured with NanoSight (NanoSight Ltd., Malvern, UK).

### Western blotting

Proteins (20 μg) were separated using bis-Tris–HCl-buffered 4%–12% gradient polyacrylamide gels (Invitrogen, Carlsbad, CA, USA) and blotted onto PVDF membranes (Millipore, Watford, UK). Membranes were blocked with 3% skim milk in TBS-T (20 mM Tris, 0.1% Tween 20, and 137 mM NaCl) at room temperature for 1 h. Primary antibodies were incubated overnight at 4°C as follows: anti-AIP1/Alix (1:250 dilution; BD Biosciences, San Jose, CA, USA), anti-CD63 (1:500 dilution; System Bioscience, Mountain View, CA, USA), anti-CD9 (1:500 dilution; System Bioscience, Mountain View, CA, USA), and anti-calnexin (1:500 dilution; Santa Cruz Biotechnology, Santa Cruz, CA, USA). Membranes were then incubated with secondary antibodies at room temperature for 1 h after washing three times with TBS-T. Secondary antibodies were used as follows: anti-mouse for AIP1/Alix (1:2,000 dilution; Invitrogen-Molecular Probes, Eugene, Oregon), anti-rabbit for CD63 and CD9 (1:2,000 dilution; System Bioscience, Mountain View, CA, USA) and anti-goat for calnexin (1:2,000 dilution; Invitrogen-Molecular Probes, Eugene, Oregon). Immuno-reactive bands were visualized using ECL reagents (Roche, Nutley, NJ, USA) and imaged using the LAS-3000 imaging system (Fuji Film, Tokyo, Japan).

### Fluorescence exosome imaging *in vitro*

A stock solution of the lipophilic tracers (Invitrogen, Carlsbad, CA, USA) DiI (red fluorescence; Ex^565 nm^, Em^594 nm^) and DiO (green fluorescence; Ex^484 nm^, Em^501 nm^) were prepared in ethanol and DMSO. Exosomes isolated from culture medium were incubated with DiO and DiI (1 μM) dye for 30 min at 37°C. Exosomes were then washed with PBS and purified using ExoQuick™. Fluorescently labeled exosomes (20 μg/mL) were used to treat tumor cells (4T1), endothelial cells (SVEC), macrophages (Raw264.7), stem cells (mBs-MSC), fibroblasts (3T3), and dentritic cells (JAWS2). Fluorescent exosomes in cells were detected using a Zeiss LSM510 META confocal imaging system (Carl Zeiss, Thornwood, CA, USA). We also constructed a pCMV driven GFP/RFP-tagged CD9 vector and imaged exosomes with confocal microscopy.

### Cell viability assay

To evaluate the cytotoxicity of DFO and exosomes in 4T1 and SVEC cells, cell viability assays were performed after 48 h of treatment with DFO (0, 200, 400, 800 μM) and exosomes (0, 200, 400, 800 μg/mL). After incubation with Cell Counting Kit-8 (CCK-8) solution for 2 h, the mean optical density (OD) at 450 nm was measured.

### Fluorescence labeling of exosomes *in vivo*

To label exosomes with Cy7, 50 μg of exosomes (total volume 100 μL) were mixed with 0.5 μg of Cy^™^7 monoNHS ester (5 μM, Sigma-Aldrich, MO, USA) for 10 min at 37°C. ExoQuick^™^ was used to purify Cy7-labeled exosomes, followed by centrifugation at 3,000 × *g* for 15 min. Cy7-labeled exosomes were imaged with the Maestro™ *in-vivo* fluorescence imaging system (Cambridge Research Instrumentation, Woburn, MA, USA).

### Tumor grafts in nude mice

All procedures involving *in vivo* mouse studies were approved by the Institutional Animal Care and Use Committee (IACUC) at Seoul National University and complied with the *Guide for the Care and Use of Laboratory Animals*. 4T1 cells (1 × 10^6^ cells) were subcutaneously transplanted in the thighs of 6-week-old male BALB/c nu/nu mice weighing 20 g on average, and tumors were grown to 10 mm in diameter.

### Fluorescence imaging *in vivo*

Cy7-labeled exosomes (200 μg) were intravenously injected in tumor xenograft mice. Exosome signals were imaged using the IVIS200 imaging system (Xenogen Corp., Alameda, CA, USA) and a CCD camera.

### Quantification of miR-210 by RT-qPCR

4T1 cells (1×10^6^ cells) were cultured in conditioned medium and treated with 400 μM DFO for 48 h. Blood from tumor-bearing mice was collected by cardiac puncture at 48 h after 400 μM DFO treatment, and exosomes were harvested from the serum. Total RNA was extracted using TRIzol reagent (Invitrogen, Carlsbad, CA, USA). Quantification of miR-210 was performed with SYBR Green real-time PCR Master Mix and Mir-X™ miRNA First-Strand Synthesis (TaKaRa, Otsu, Japan) according to the manufacturer’s instruction. U6 was used as a housekeeping gene to standardize the initial miRNAs from a sample. Data are presented as fold downregulation or upregulation. Fold value = 2^-ΔΔCt^, where ΔΔCt = (Ct of gene of interest, treated-Ct of housekeeping gene, treated)-(Ct of gene of interest, control-Ct of housekeeping gene, control), and Ct was the number of threshold cycles. Primer sequences were used as follows: miR-210 forward 5′-CTGTGCGTGTGACAGCGGCTGA-3′, HIF-1α forward 5′-GCACAGGCCACATTCACG-3′, and U6 forward 5′-TGGCCCCTGCGCAAGGATG-3′.

### Effects of hypoxic exosomes in SVEC cells

To confirm the effects of hypoxic exosomes, SVEC cells were treated with hypoxic exosomes from 4T1 cells (400 μg/mL) for 48 h. Wound healing assay was performed and capillary-like structures were confirmed by microscopy.

### Imaging of miR-210 *in vitro*

4T1-luc2/miR210 cells (1×10^5^ cells) were seeded in 24-well plates. Cells were treated with DFO (400 μM) or exosomes (400 μg) isolated from cells treated with DFO (400 μM). After 48 h, cells were treated with 100 μL of luciferin (0.3 μg/μL) before bioluminescence imaging. Images were obtained using the IVIS200 imaging system equipped with a CCD camera (Xenogen Corp., Alameda, CA, USA). Bioluminescent images were analyzed using LIVINGIMAGE V. 2.50.1 software (Xenogen Corp., Alameda, CA, USA).

### Luciferase assays *in vitro*

Luciferase enzyme assays were performed using luciferase assay kits (Applied Biosystems, Carlsbad, CA, USA). Cells were plated and treated with DFO or exosomes, as previously described. After 48 h, the wells were washed twice with PBS and lysis solution was added to each well. Cell lysates were then transferred to a microplate. Bioluminescence was measured using a Wallac 1420 VICTOR3 V plate reader (PerkinElmer Life and Analytical Sciences, Shelton, CT, USA).

### Imaging of miR-210 *in vivo*

Mice were injected intratumorally with PBS (in the left thigh) and 400 μM of DFO (in the right thigh). In the exosome treatment group, mice were injected intratumorally with exosomes (400 μg/mL) in the right thigh. After 48 h, mice were intraperitoneally injected with 100 μL of the luciferase substrate luciferin (30 μg/μL). Mice were anesthetized with isoflurane and images were obtained using the IVIS200 imaging system, as described.

### Immunohistochemistry (IHC)

Tumor tissues were fixed in 3.7% paraformaldehyde for 24 h and embedded with paraffin, prepared as 4-μm sections, and mounted on slides. Antigens were retrieved by boiling in citrate buffer (DakoCytomation, Glostrup, Denmark) for 5 min. Primary antibodies were incubated overnight at 4°C as follows: anti-luciferase (1:500 dilution; Abcam, Cambridge, UK), anti-HIF-1α (1:100 dilution; Novus Biologicals, Littleton, CO, USA), and anti-Ephrin-A3 (1:500 dilution; Santa Cruz Biotechnology, Santa Cruz, CA, USA). Secondary antibodies were used as follows: biotinylated anti-goat for luciferase (1:500 dilution; Dako, Glostrup, Denmark) and biotinylated anti-mouse for HIF-1α and Ephrin-A3 (1:500 dilution; Dako, Glostrup, Denmark). An avidin–biotin peroxidase complex was used to amplify the signal, followed by development using DAB and counterstaining with hematoxylin.

### Deparaffinization and protein extraction

Deparaffinization and protein extraction were performed using Qiagen’s Qproteome FFPE Tissue Kit (Qiagen, Hilden, Germany). All FFPE samples were fixed in 3.7% paraformaldehyde for 24 h and used for protein extraction. Six slides each with 4 μm sections and areas of up to 100 mm^2^ were randomly selected. Briefly, paraffin tissue slides were removed by immersion in xylene for 10 min at room temperature. Tissues were rehydrated with a graded ethanol series (100%, 95%, and 70%) followed by double-distilled water. Using a needle, areas of tissue were excised and transferred to 1.5-mL collection tubes. Extraction buffer and β-mercaptoethanol (Sigma-Aldrich, St Louis, MO, USA) were added to the tube at the recommended volume and incubated at 100°C for 20 min in order to reduce disulfide bonds, followed by incubation at 80°C for 2 h to ensure maximal protein extraction. Finally, the product was centrifuged for 15 min at 14000 × *g*, and protein extracts were stored at -20°C. Protein concentration was measured using the BCA protein assay kit (Pierce, Rockford, IL, USA) according to the manufacturer’s protocol.

### Western blot from FFPE tissue

Proteins (50 μg) were extracted from FFPE tissues and separated as mentioned. Primary antibodies were incubated overnight at 4°C as follows: anti-Ephrin-A3 (1:50 dilution; Santa Cruz Biotechnology, Santa Cruz, CA, USA), anti-PTP1B (1:200 dilution; Abcam, Cambridge, UK), and anti-VEGF (1:200 dilution; Abcam, Cambridge, UK). Secondary antibodies were used as follows: anti-rabbit for Ephrin-A3, PTP1B, and VEGF (1:2,000 dilution, Cell Signaling Technology, Danvers, MA, USA). Beta-actin was used as a loading control.

### Statistical analysis

All results are presented as the mean ± SD. Student's unpaired t-test was used to determine statistical significance, and P-values < 0.05 were considered statistically significant.

## SUPPLEMENTARY FIGURES


