# Supplementary material for: Visualization of exosome-mediated miR-210 transfer from hypoxic tumor cells
Source: Oncotarget. 2016 Dec 27;8(6):9899–910. doi: 10.18632/oncotarget.14247 (PMC5354779; doi:10.18632/oncotarget.14247)
Supplement: Supplementary file 1 [file oncotarget-08-9899-s001.pdf]

Visualization of exosome-mediated miR-210 transfer from hypoxic tumor cells

SUPPLEMENTARY FIGURES

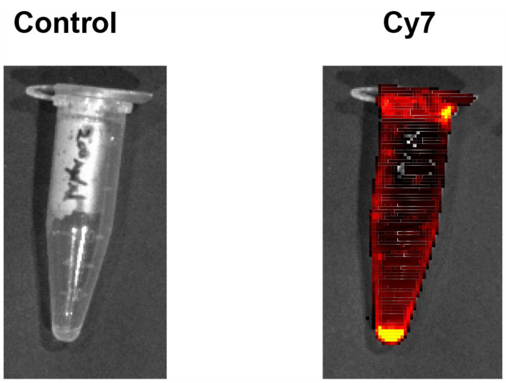

Supplementary Figure 1: Cy7-labeled exosomes.

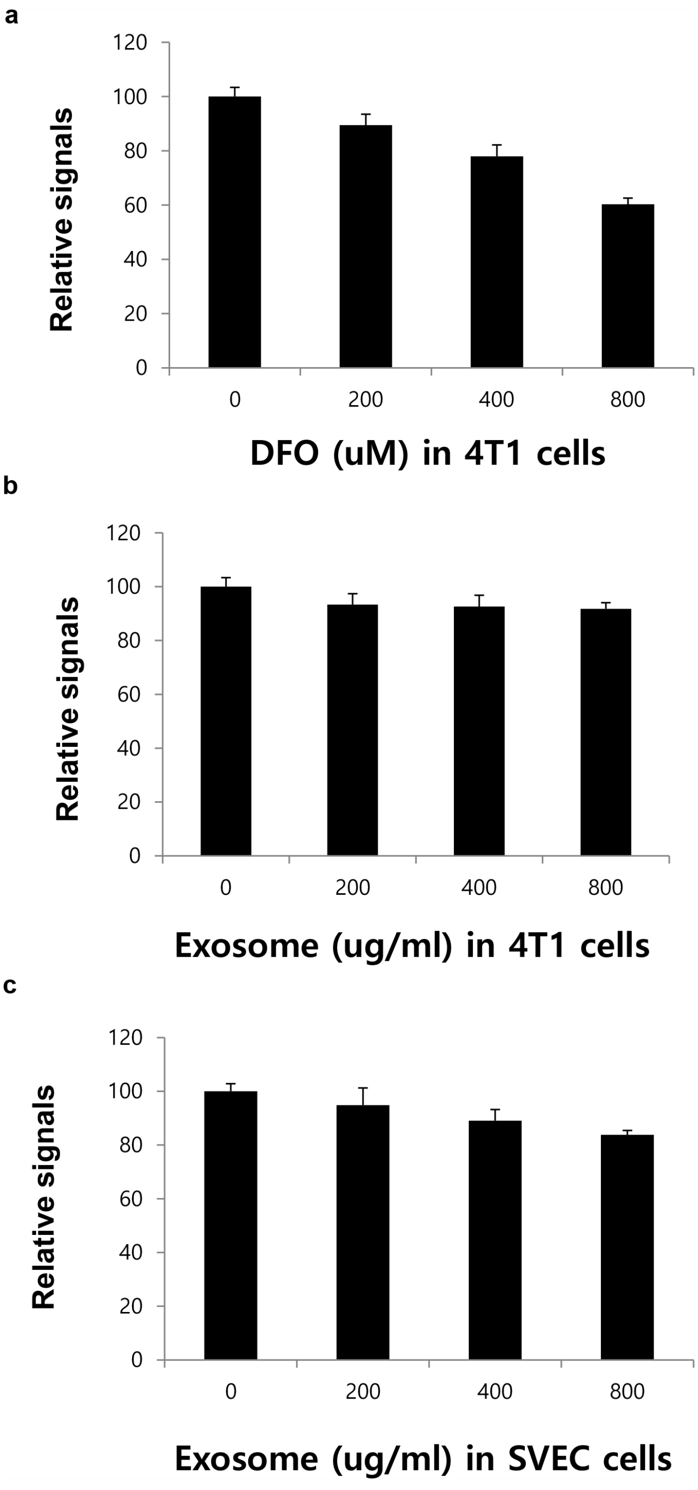

Supplementary Figure 2: Cell viability assays. a. 4T1 cells were treated with DFO for 48 h. **b, c.** 4T1-luc2/miR-210 and SVEC-luc2/miR-210 cells were treated with hypoxic exosomes and for 48 h. Hypoxic exosomes were not cytotoxic, even at high concentrations in both 4T1 and SVEC cell lines.

a

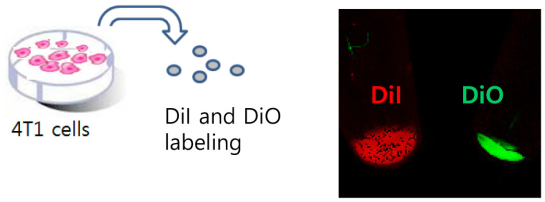

b

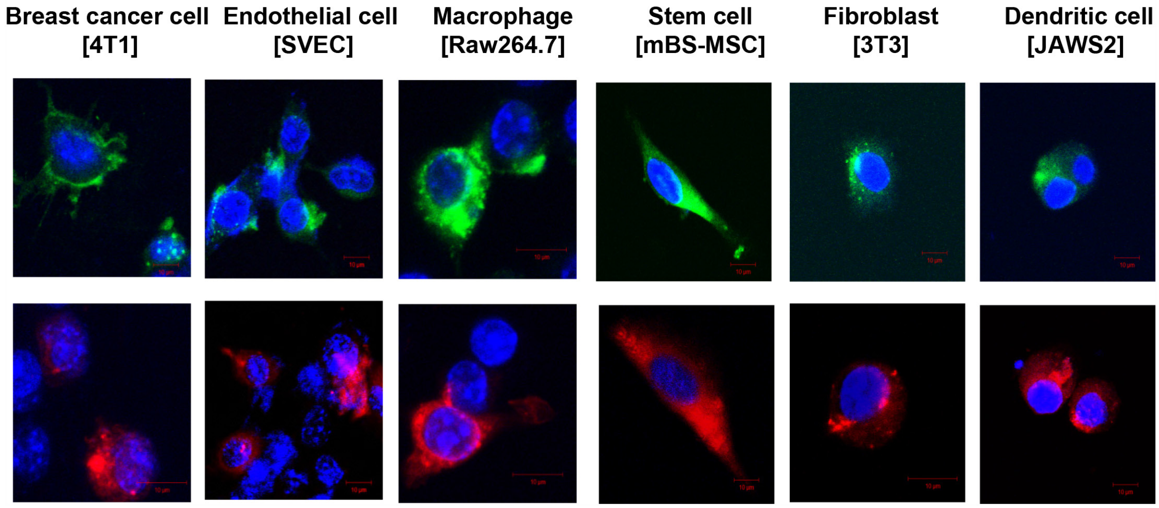

Supplementary Figure 3: Exosome uptake of cells in the tumor microenvironment. Exosomes isolated from 4T1 cells were labeled with the lipophilic tracers DiI or DiO. **a.** Exosome pellets. **b.** Exosome uptake in 4T1, SVEC, Raw264.7, mBs-MSC, 3T3, and JAWS2 cells.

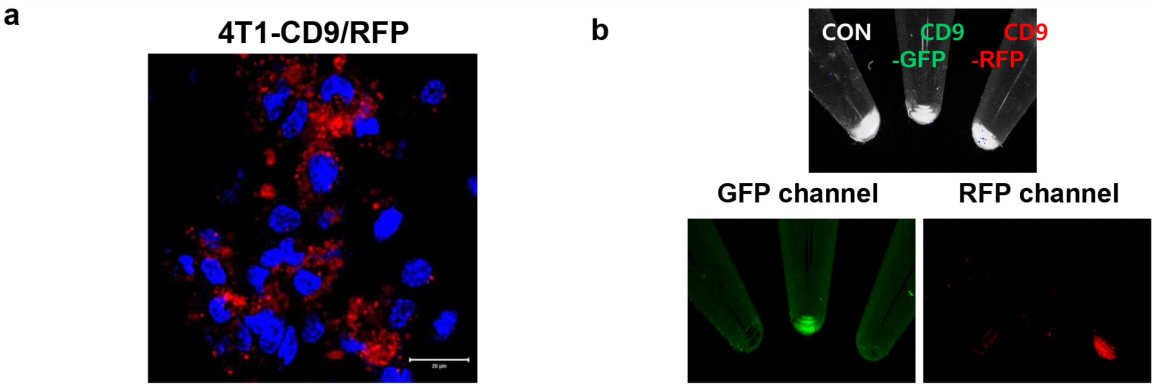

Supplementary Figure 4: Exosomes expressing CD9/RFP reporter vector. a. Confocal microscopy of CD9/RFP expressing 4T1 cells. b. Exosome pellets isolated from 4T1-CD9/RFP and 4T1-CD9/GFP cells.

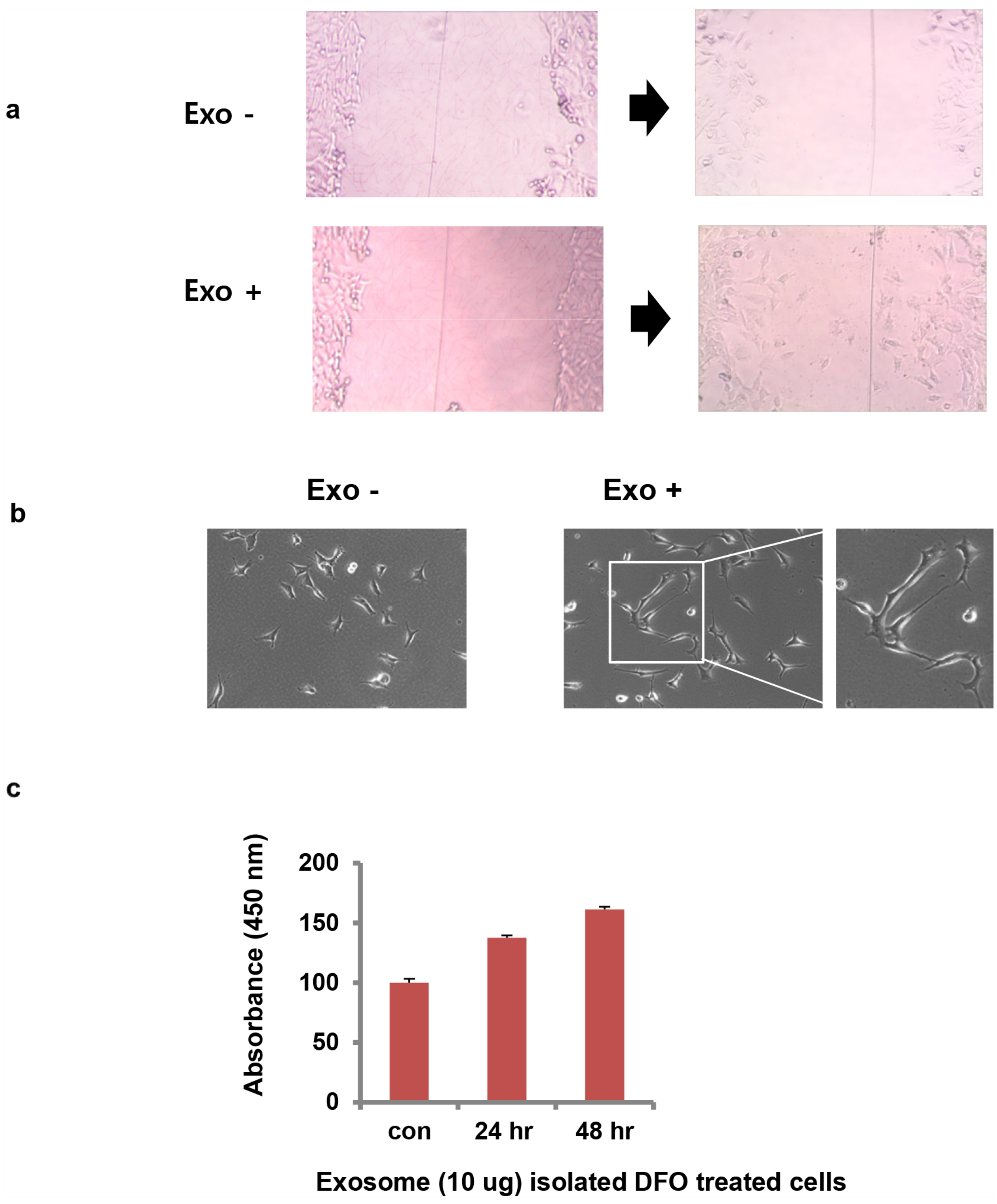

Supplementary Figure 5: Effects of hypoxic exosomes in SVEC cells. a. Wound healing assay showed that migration of SVEC cells was increased in exosome-treated cells. **b.** Capillary-like structures were also increased in exosome-treated SVEC cells. **c.** Proliferation was increased in exosome-treated SVEC cells.
